# User Evaluation of the Swedish Patient Accessible Electronic Health Record: System Usability Scale

**DOI:** 10.2196/24927

**Published:** 2021-07-27

**Authors:** Maria Hägglund, Isabella Scandurra

**Affiliations:** 1 Healthcare Sciences and e-Health Department of Women's and Children's Health Uppsala University Uppsala Sweden; 2 School of Business Örebro University Örebro Sweden

**Keywords:** usability, system usability scale, evaluation, patient accessible electronic health records, open notes, patient portals

## Abstract

**Background:**

Transparency is increasingly called for in health care, especially, when it comes to patients’ access to their electronic health records. In Sweden, the e-service Journalen is a national patient accessible electronic health record (PAEHR), accessible online via the national patient portal. User characteristics and perceived benefits of using a PAEHR influence behavioral intention for use and adoption, but poor usability that increases the effort expectancy can have a negative impact. It is, therefore, of interest to explore how users of the PAEHR Journalen perceive its usability and usefulness.

**Objective:**

The aim of this study was to explore how the users of the Swedish PAEHR experience the usability of the system and to identify differences in these experiences based on the level of transparency of the region.

**Methods:**

A survey study was conducted to elicit opinions and experiences of patients using Journalen. The data were collected from June to October 2016. The questionnaire included questions regarding the usability of the system from the System Usability Scale (SUS). The SUS analysis was the focus of this paper. Analysis was performed on different levels: nationally looking at the whole data set and breaking it down by focusing on 2 different regions to explore differences in experienced usability based on the level of transparency.

**Results:**

During the survey period, 423,141 users logged into Journalen, of which 2587 unique users completed the survey (response rate 0.61%). The total mean score for all respondents to the SUS items was 79.81 (SD 14.25), which corresponds to a system with good usability. To further explore whether the level of transparency in a region would affect the user’s experience of the usability of the system, we analyzed the 2 regions with the most respondents: Region Uppsala (the first to launch, with a high level of transparency), and Region Skåne (an early implementer, with a low level of transparency at the time of the survey). Of the participants who responded to at least 1 SUS statement, 520 stated that they had received care in Region Skåne, whereas 331 participants had received care in Region Uppsala. Uppsala’s mean SUS score was 80.71 (SD 13.41), compared with Skåne’s mean of 79.37 (SD 13.78).

**Conclusions:**

The Swedish national PAEHR Journalen has a reasonably good usability (mean SUS score 79.81, SD 14.25); however, further research into more specific usability areas are needed to ensure usefulness and ease of use in the future. A somewhat higher SUS score for the region with high transparency compared with the region with low transparency could indicate a relationship between the perceived usability of a PAEHR and the level of transparency offered, but further research on the relationship between transparency and usability is required.

## Introduction

Transparency, including the possibility for patients to gain insight into one’s own medical information, is increasingly called for in health care, especially, when it comes to patients’ access to their electronic health records (EHRs) [1]. Many countries (eg, Finland, France, Norway, Australia, Denmark, Canada, United Kingdom, and Sweden) have, in the past, implemented or are now currently implementing patient accessible electronic health records (PAEHRs) [2]. In some countries, these are local implementations at a specific hospital or region, whereas others have national solutions. Differences in strategies and approaches have affected the uptake and impact, and the implementation progress has, in several countries, been slow due to legal constraints [3,4] and concerns about security and privacy among health care professionals [5-7]. A PAEHR often includes the clinical notes written by different health care professionals, as well as other parts of the EHR (eg, lab results, referrals, and medications).

In the United States, the OpenNotes initiative focuses on providing patients access to their notes, specifically. OpenNotes began as a pilot evaluation project that included 105 volunteer primary care physicians and their 19,000 patients [8,9]. The initiative started in 2010 and has since spread throughout the United States [10]. On April 5, 2021, a new federal rule required US health care providers to allow patients access to all the health information in their EHR [11,12]. This new rule mandates rapid, full access to test results, medication lists, referral information, and clinical notes in electronic formats, by request.

In Sweden, the e-service Journalen is a national PAEHR, accessible online via the national patient portal called 1177.se [13]. The PAEHR service accesses the EHR information from most of the various EHR systems used throughout Swedish health care organizations, via a national health information exchange platform [14,15]. Hence, patients have one access point for all their health record information regardless of (1) how many health care providers they have visited and (2) which EHR system their health care providers use [13]. Since the first Swedish region began providing their inhabitants online access to their health records in 2012, all the other regions have connected to the national infrastructure and the PAEHR Journalen. This was not the case from the beginning, though, and the last of the 21 regions connected only in April 2018. In addition, different regions made different choices about how much of their information would be made available to patients; for example, patients receiving care in one region could gain access to both their lab results and notes, whereas patients receiving care in a different region might only be able to access the notes [13,16].

A growing literature on patients’ experiences of accessing their records online reports positive outcomes [10,16]. Patients who read their notes have reported a better understanding of their care plans [10], a sense of greater control over their care [10,16], an improved adherence to medication [17], improved communication with and trust in their clinicians [16,17], and a sense that their care is safer [18].

Despite these benefits, adoption and use can be low [19], and several studies have explored factors that influence adoption [20,21]. User characteristics and perceived benefits of using a PAEHR might influence behavioral intention for use and adoption, but poor usability that increases the effort expectancy can also have an impact. It is, therefore, of interest to further explore how users of the PAEHR Journalen perceive its usability and usefulness; the latter is especially important, considering the differences in the levels of transparency regarding patients’ health information in different regions.

In this study, we analyzed data on usability issues from a national survey conducted among patients who use the PAEHR Journalen. A first analysis of the main results from the survey was published in 2018 [16] and contains an overview of the full survey. Here, we focused only on the usability-related questions as well as some demographic data of the participants. At the time of the study (June to October 2016), not all regions allowed patients access to their records through Journalen, and, among those who did, the level of transparency of this information varied [16]. Table 1 represents an overview of what types of clinical content the health care providers had chosen to allow access to at the time of data collection for this study.

The aim of this study was to explore how the users of the Swedish PAEHR experience the usability of the system and to identify differences in these experiences based on the level of transparency regarding patients’ health information for the region.

**Table 1:** Overview of core types of clinical content the health care providers (21 regions and 1 private care provider) had chosen to allow access to at the time of data collection for this study (adapted from [16]).

Care provider	Content provided								
	Medical notes (18/22, 82%)	Diagnoses (15/22, 68%)	Lab results (8/22, 36%)	Medications (7/22, 32%)	Immunizations (7/22, 32%)	Referrals (5/22, 23%)	Access to log lists (3/22, 14%)	Psychiatry notes (2/22, 9%)	Total content available
Blekinge	✓	✓	✓	✓					4
Dalarna	✓					✓			2
Gotland									0
Gävleborg									0
Halland	✓	✓	✓		✓				4
Jämtland/ Härjedalen									0
Jönköping	✓	✓		✓					3
Kalmar	✓	✓	✓	✓					4
Kronoberg	✓	✓						✓	3
Norrbotten	✓			✓	✓				3
Skåne	✓	✓						✓	3
Stockholm	✓	✓	✓						3
Södermanland	✓	✓							2
Uppsala	✓	✓	✓	✓		✓	✓		6
Värmland	✓	✓	✓		✓	✓	✓		6
Västerbotten	✓	✓		✓					3
Västernorrland									0
Västmanland	✓					✓	✓		3
VGR	✓	✓							2
Örebro	✓	✓	✓	✓	✓				5
Östergötland	✓	✓	✓	✓	✓	✓			6
Capio (private care provider)	✓	✓	✓						3

## Methods

### Study Design

A survey study was conducted to collect opinions and experiences of patients using Journalen. The data were collected from June to October 2016, after ethical approval of the research was granted by the Regional Ethical Review Board in Uppsala, Sweden (EPN 2016/129). Participants were recruited through the national PAEHR Journalen. When patients logged into Journalen, they received a request for voluntary survey participation together with information about the study. Thus, only active users of Journalen were invited to participate.

### Data Collection

#### Survey Preparation

An anonymous questionnaire was designed covering different topic areas with a total of 24 questions in Swedish (see the full questionnaire in [16]), including questions regarding the usability of the system using the System Usability Scale (SUS) [22].

The usability and technical functionality of the electronic questionnaire had not been tested before fielding the questionnaire. However, participants received information about whom to contact in case of technical issues. The SUS has been validated and used in many studies [23].

The collected data were managed by the eHealth service provider Inera AB, in accordance with the Regional Ethical Review Board’s approval. The survey data were stored in the same database system as the PAEHR Journalen, meaning that the collected data, including patient IDs, had the same security protection as all patient information handled in the PAEHR. A patient ID was stored during the collection period to ensure that patients had not left duplicate responses. When the collection period was completed, the patient ID was removed and all stored information was anonymized. The anonymized dataset was exported to researchers for analysis.

#### The System Usability Scale

The SUS [22] is a simple, 5-point Likert scale that provides a global view of subjective assessments of usability, which was developed as a fast and efficient method to collect an overview of the usability of a system [24]. Benefits of the SUS tool include that it is technologically agnostic (ie, it can be used for many different types of information technology systems), that it is quick and easy to use for both participants and researchers, that it provides a single score on a scale that is easy to understand, and that it is cost efficient due to its state of nonpropriety [24]. The SUS consists of 10 statements that were slightly modified and translated to Swedish for this study (Table 2).

**Table 2:** The System Usability Scale statements^a^ and our modifications.

Item	SUS^b^ statement	Modified statement	Statement in Swedish
1	I think that I would like to use this system frequently.	I think that I would like to use Journalen regularly.	Jag tror att jag vill använda ”Journalen” regelbundet.
2	I found the system unnecessarily complex.	I found Journalen unnecessarily complex.	Jag anser att ”Journalen” är mer komplicerad än vad den behöver vara.
3	I thought the system was easy to use.	I thought Journalen was easy to use.	Jag anser att ”Journalen” är lätt att använda.
4	I think that I would need the support of a technical person to be able to use this system.	I think that I would need the support of a technical person to be able to use Journalen.	Jag tror att jag skulle behöva personlig teknisk support för att kunna använda ”Journalen.”
5	I found the various functions in this system were well integrated.	I found the various functions in the system were well integrated.	Jag anser att de olika funktionerna i ”Journalen” fungerar väl tillsammans.
6	I thought there was too much inconsistency in this system.	I thought there was too much inconsistency in this system.	Jag anser att det finns många delar i ”Journalen” som inte är konsekventa.
7	I would imagine that most people would learn to use this system very quickly.	I would imagine that most people would learn to use Journalen very quickly.	Jag tror att de flesta skulle kunna lära sig att använda ”Journalen” ganska snabbt.
8	I found the system very cumbersome to use.	I found Journalen very cumbersome to use.	Jag anser att ”Journalen” är besvärlig att använda.
9	I felt very confident using the system.	I felt very confident using Journalen.	Jag känner mig väldigt säker och trygg (på vad jag gör) när jag använder ”Journalen.”
10	I needed to learn a lot of things before I could get going with this system.	I needed to learn a lot things before I could get going with Journalen.	Jag behöver lära mig ganska mycket innan jag kan börja använda ”Journalen.”

^a^Responses were measured with a 5-point Likert scale.

^b^SUS: System Usability Scale.

### Data Analysis

#### Main Analyses

Overall, 2587 patients from 21 regions completed the survey. The number of participants for each region varied. Notably, it was not possible to statistically verify whether the number of participants was at an adequate level to provide more than tentative region-wise and group-wise comparisons. Only completed questionnaires have been analyzed, as the answers were stored in the database only when the participant chose to submit the survey on the last page. However, the SUS items were not mandatory to respond to, and, therefore, the total number of answers for each SUS item varied (Table 3). In addition, 48 participants did not answer any of the SUS items and were excluded from further analysis, leaving 2539 people who answered at least 1 SUS item. Item 1 had the most answers (n=2507), whereas item 6 had the fewest (n=2459). Some free-text comments also indicated that item 6 was difficult to understand for some of the participants.

Rather than excluding questionnaires with missing SUS answers, we have chosen to substitute a neutral (eg, “neither agree nor disagree”) response for the missing items. Since individual items on the SUS score are not necessarily meaningful themselves, this was a feasible approach to make sure that we did not tilt the results to one of the extremes when simply excluding a response.

In this paper, we focused on the SUS questions, which were analyzed according to the SUS method. Questions regarding demographics and perceived usefulness were also included in the analysis (for these questions, we used all survey responses, not excluding those who did not respond to the SUS statements). The analysis was completed on different levels: (1) nationally looking at the whole data set and (2) breaking it down by focusing on 2 different regions to explore differences in experienced usability based on the level of transparency. The 2 different regions were Uppsala (the first to launch, with a high level of transparency), and Skåne (an early implementer, with a low level of transparency at the time of the survey).

**Table 3:** The number of answers for each System Usability Scale item (N=2539).

Item	Modified SUS^a^ item	Total answers
1	I think that I would like to use Journalen regularly.	2507
2	I found Journalen unnecessarily complex.	2476
3	I thought Journalen was easy to use.	2498
4	I think that I would need the support of a technical person to be able to use Journalen.	2471
5	I found the various functions in the system were well integrated.	2481
6	I thought there was too much inconsistency in this system.	2459
7	I would imagine that most people would learn to use Journalen very quickly.	2479
8	I found Journalen very cumbersome to use.	2462
9	I felt very confident using Journalen.	2482
10	I needed to learn a lot things before I could get going with Journalen.	2448

^a^SUS: System Usability Scale.

#### SUS Analysis

We decided to include all the answers to the SUS items in our calculation, in which participants responded to at least one SUS item, despite some participants not answering all items. We calculated the individual analysis for each participant’s SUS score, and the median and mean values for the entire population. The final scores for the SUS can range from 0 to 100, where higher scores indicate better usability. Because the statements alternate between positive and negative, care must be taken when scoring the survey. To calculate the SUS score, each item’s score contribution (ranging from 0-4) must be calculated. For items 1, 3, 5, 7, and 9, the score contribution is the scale position minus 1. For items 2, 4, 6, 8, and 10, the contribution is 5 minus the scale position. For participants who missed 1 or more SUS question, we chose to substitute a neutral (“neither agree nor disagree”) response for missing items.

The score contributions for each item were then added together and multiplied by 2.5 to achieve the final score [22]. According to Bangor and colleagues’ [24] thorough evaluation of the SUS, a system needs to score above 70 to be considered at least passable. Better systems will score in the high 70s to high 80s, and scores over 90 indicate a truly superior system [24]. The authors also argued that any system that scores below 70 would require further usability testing and continued improvement.

We made 3 separate SUS calculations: (1) a calculation of all the individual answers, (2) a calculation of only answers from participants from Uppsala, and (3) a calculation of only answers from participants from Skåne.

## Results

During the survey period, 423,141 users logged into Journalen, of which 2587 patients completed the survey (of unique users that logged in, response rate 0.61%). Of all respondents, 62.97% (1629/2587) identified as women and 30.85% (798/2587) as men; 0.39% (10/2587) of respondents chose “other,” and 5.80% (150/2587) did not answer this question. According to use statistics provided by Inera AB (the company providing Journalen and the national patient portal [25]), this reflects the gender distribution of the users in general (in 2016, 60% women and 40% men). Of all respondents, 39.81% (1030/2587) stated that they were working or had been working within health care, and 54.54% (1411/2587) stated that they had no professional relation to health care; 5.64% (146/2587) of respondents did not answer this question. Participants had a higher education level than the general population [16]. Among our participants, 60.57% (1487/2455) had higher education, whereas only 42% of the general Swedish population does [26]. We cannot tell whether this is because users of Journalen are well educated or that people with a higher education represent a subgroup of users who are more inclined to answer a survey. Unfortunately, no data on the general education levels of Journalen users exist.

To sum up, the survey results regarding user characteristics on a national level indicate that most participants were women and that the majority had studied at least 3 years of higher education. In addition, results indicate that many users of Journalen were both patients and medical professionals, at various points in their lives.

In Moll and colleagues’ [16] overview of the survey results, details of the participants’ views of the usefulness and benefits of accessing their health records online are presented in more detail. Overall, patients who answered the survey were positive toward Journalen (Table 4). Participants were asked to rate on a 5-point Likert scale to what extent they agreed to the more general statements, “I think that access to one’s medical records online is generally a good reform,” and “I think that access to Journalen is good for me.” Of all participants, >96% (2454/2541, 96.58% and 2455/2528, 97.11%, for the respective questions) had a positive attitude toward Journalen, answering with either “completely agree” or “partly agree.”

**Table 4:** Participants’ attitudes toward patients’ access to their medical records online.

Item		Value, n (%)
**I think access to one’s medical records online is generally a good reform.**		2541^a^
	Do not agree at all	26 (1.02)
	Do not agree	23 (0.91)
	Neutral	38 (1.50)
	Partly agree	302 (11.89)
	Completely agree	2152 (84.69)
**I think that access to Journalen is good for me.**		2528^a^
	Do not agree at all	19 (0.75)
	Do not agree	15 (0.59)
	Neutral	39 (1.54)
	Partly agree	199 (7.87)
	Completely agree	2256 (89.24)

^a^Some participants did not answer all questions. Therefore, the total for each variable category differs.

However, a positive attitude toward accessing one’s health records does not say much about the usability of the system, and, therefore, we also present the results of the SUS analysis. Results of the analysis of the SUS questions are first described on a national level. Table 5 presents the results of all participants for the SUS items in the survey, including neutral responses replacing missing answers for participants who responded to at least 1 SUS item.

**Table 5:** Results of the System Usability Scale items for all participants, on a national level (N=2539)^a^.

SUS^b^ analysis item	Value per 5-point Likert scale response^c^, n (%)				
	1	2	3	4	5
I think that I would like to use Journalen regularly.	37 (1.46)	39 (1.54)	182 (7.16)	529 (20.83)	1752 (69.00)
I found Journalen unnecessarily complex.	915 (36.04)	723 (28.48)	577 (22.73)	244 (9.61)	80 (3.15)
I thought Journalen was easy to use.	40 (1.58)	70 (2.76)	285 (11.22)	785 (30.92)	1359 (53.53)
I think that I would need the support of a technical person to be able to use Journalen.	1843 (72.59)	391 (15.40)	205 (8.07)	66 (2.60)	34 (1.33)
I found the various functions in the system were well integrated.	62 (2.44)	171 (6.73)	720 (28.36)	831 (32.73)	755 (29.74)
I thought there was too much inconsistency in this system.	550 (21.66)	466 (18.35)	1120 (44.11)	305 (12.01)	98 (3.86)
I would imagine that most people would learn to use Journalen very quickly.	29 (1.14)	103 (4.06)	355 (13.98)	1140 (44.90)	912 (35.92)
I found Journalen very cumbersome to use.	1587 (62.50)	531 (20.91)	278 (10.95)	96 (3.78)	47 (1.85)
I felt very confident using Journalen.	54 (2.13)	37 (1.46)	275 (10.83)	602 (23.71)	1571 (61.87)
I needed to learn a lot things before I could get going with Journalen.	1790 (70.50)	421 (16.58)	235 (9.26)	64 (2.52)	29 (1.14)

^a^The appropriate number of neutral responses were added to replace missing responses for each item, in order to not skew results due to missing items.

^b^SUS: System Usability Scale.

^c^From 1 (“Do not agree at all”) to 5 (“Completely agree”).

**Figure 1:**
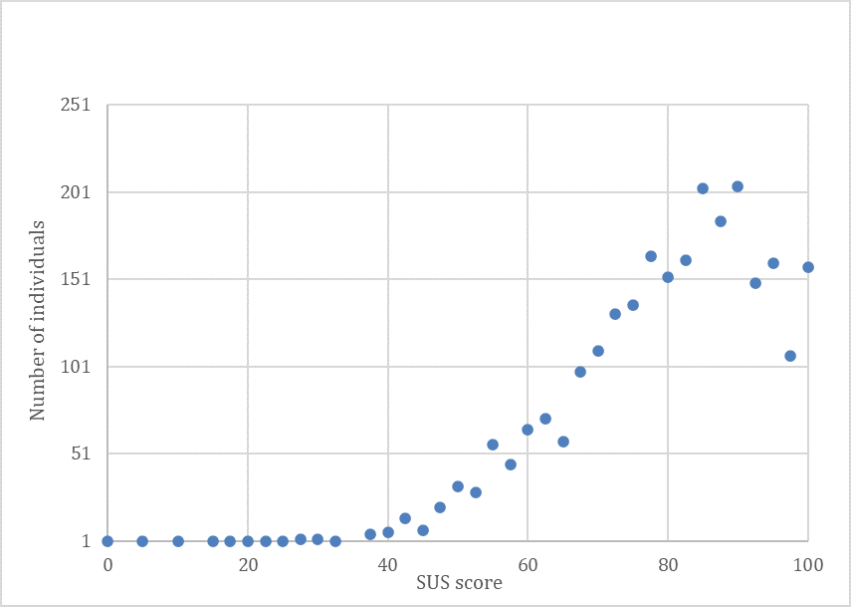
Individual System Usability Scale scores. SUS: System Usability Scale.

For all participants, the total mean score for the SUS scale was 79.81 (SD 14.25), which would, according to Bangor and colleagues [24], qualify as a successful system. The median score was 82.5, and the distribution of individual answers is plotted in Figure 1. The scores covered the entire range, from 0 (1 person) to 100 (158 people), but the majority of individuals scored above 60.

Each item can have a score contribution between 0 and 4. Most of the items scored above 3; however, 3 questions stood out with score contributions below 3 (Table 6). All 3 questions that score below 3 related to the complexity of the system and whether functions are well integrated in the system.

**Table 6:** System Usability Scale score contribution of individual items.

SUS^a^ analysis item	Score
I think that I would like to use Journalen regularly.	3.54
I found Journalen unnecessarily complex.	2.85
I thought Journalen was easy to use.	3.32
I think that I would need the support of a technical person to be able to use Journalen.	3.55
I found the various functions in the system were well integrated.	2.81
I thought there was too much inconsistency in this system.	2.42
I would imagine that most people would learn to use Journalen very quickly.	3.10
I found Journalen very cumbersome to use.	3.38
I felt very confident using Journalen.	3.42
I needed to learn a lot things before I could get going with Journalen.	3.53

^a^SUS: System Usability Scale.

To further explore whether the level of transparency in a region would affect the user’s experience of the usability of the system, we made additional SUS analyses based on the 2 regions with the most participants: Region Uppsala (the first to launch, with a high level of transparency) and Region Skåne (an early implementer, with a low level of transparency at the time of the survey). Overall, 692 participants stated that they had received care in Region Skåne, of which 520 responded to at least 1 SUS item and were included in this analysis. However, 520 participants stated that they had received care in Region Uppsala, of which 331 had responded to at least 1 SUS item. Analysis of Region Skåne responses yielded a final score of 79.37, whereas Uppsala’s result was 80.71. The results in mean SUS score were close enough to indicate that no major difference can be seen on the SUS scale based on the level of transparency in the implementation of the PAEHR. A two-tailed *t* test with equal variance yielded a *P* value of .16, indicating that the difference was not statistically significant at the standard 5% significance level.

In addition to the SUS, we also asked a question specifically focused on navigation, more precisely, on the participants’ experiences of locating Journalen in the national patient portal. This issue had been brought up as a concern previously; since the national patient portal contains many eHealth services, there were worries that patients might not find Journalen. The majority of the participants (1974/2451, 80.53%) did not have trouble locating the link in the patient portal, whereas 233/2451 (9.51%) expressed difficulty finding Journalen. 

## Discussion

To summarize, the results indicate that the users of the Swedish PAEHR Journalen rate the service high (79.81) on the System Usability Scale, yet questions relating to consistency and complexity scored lower. Before discussing the results in more detail, we address some methodological limitations of this study.

### The System Usability Scale

The SUS does not help identify specific usability issues or provide detailed information on effectiveness or efficiency of the system that is evaluated. For an in-depth usability evaluation, usability testing or other forms of usability evaluations would be necessary. However, to gain an overall understanding of the level of usability of a system, the SUS can be useful [24]. In this study, we chose to use SUS items as parts of a more extensive survey to achieve an overall understanding of the usability of the current version of the Swedish PAEHR Journalen. An SUS score could also be used as a baseline analysis for further evaluations of the PAEHR, particularly, when changes to PAEHR’s user interface or content have been implemented.

### Limitations of the Survey

The survey distribution may have created a bias in the study, which needs to be considered when interpreting the results. The survey was distributed through the national patient portal and was only accessible once someone logged into Journalen. This was intentional, as the main aim of the study was to explore the experiences of people who had used the e-service. This does, however, mean that only users with the skills and competence to access Journalen were able to answer the survey. If we, instead, had recruited people to represent the entire Swedish population, the results may have been different. In addition, a user who had previously tried using Journalen but did not find it very useful or usable might not have returned at all and would therefore not have found the survey. Hence, our results are likely biased toward more positive users.

In addition, it is not possible to determine whether the participants of our survey are representative of all users of Journalen. As in most survey studies, the participants form a small sample of all possible users, and many more users than those who answered the survey logged into Journalen during the 5 months that the survey was open. We do not know whether the demographic distribution is representative. Our survey participants have a higher education level than the general population, but, unfortunately, we do not know the education levels of all Journalen users. Among our participants, 60.57% (1487/2455) had higher education, whereas only 42% of the general Swedish population does [26]. We cannot tell whether this is because users of Journalen are typically well educated; it may also be that the well-educated users are more likely to answer a survey. An interesting future study would be to explore further whether user education level and eHealth literacy would impact the score on the System Usability Scale.

A high proportion of our participants also had experiences of working in health care. We can only hypothesize as to why this is the case; perhaps health care professionals are more likely to use eHealth services themselves. In future studies, it would also be of interest to see if health care professionals’ assessments of the usability of the PAEHR differ from other users, and, if so, how these assessments differ.

Finally, in this study, we have not further analyzed differences in characteristics between users who scored low on the SUS scale and those who scored higher. If we can distinguish characteristics of the low scorers, the needs of these users could be targeted in future redesigns of the PAEHR.

### Information Access Through a National Solution

Since the Swedish PAEHR Journalen is built on a national platform, its design and functionality are the same for all users throughout Sweden. However, the clinical content or information that is accessible to patients varies depending on the local regulations in each region. Here, we had an opportunity to explore whether this level of transparency in a region would have an impact on the usability experienced by the end user.

Uppsala (a high transparency area) scored 80.71 and Skåne (a low transparency area) scored 79.37 on the SUS scale, with more than 1-point difference between the 2 groups. This might possibly be due to a lower level of transparency causing frustration among the users in Skåne. However, we cannot answer this question based on these results; many other factors could influence these results.

### Conclusions

We conclude that the participants of this survey rated usability of the Swedish national PAEHR Journalen high (scoring 80 on the SUS); however, further research into more specific usability areas is needed to ensure usefulness and ease of use in the future. A somewhat higher SUS score for the region of Uppsala as compared with Skåne could indicate a relationship between the perceived usability of a PAEHR and the level of transparency regarding patients’ health information, but these differences in usability could also be related to other regional differences in the implementations of the PAEHR.

## Abbreviations

EHR: electronic health record
PAEHR: patient accessible electronic health record
SUS: System Usability Scale
